# Impact on urinary incontinence after management of complications related to a retropubic midurethral sling

**DOI:** 10.1007/s00192-023-05600-7

**Published:** 2023-07-20

**Authors:** Caroline Juhl, Mette Holberg Thimm, Karin Glavind

**Affiliations:** Department of Gynecology and Obstetrics, Aalborg University Hospital, Reberbansgade 15, 9000 Aalborg, Denmark

**Keywords:** Complications, Complication treatment, Stress urinary incontinence, Tension-free vaginal tape, Midurethral sling

## Abstract

**Introduction and hypothesis:**

The most common complications to midurethral sling (MUS) operations for stress urinary incontinence are postoperative urinary retention (POUR), vaginal MUS exposure, and urgency. They are well described but consensus regarding their management is missing. An evaluation of the treatment of POUR, exposure and urgency after the MUS procedure in our department was implemented. Incontinence status after treatment of complications was evaluated.

**Methods:**

A review of the medical records of women undergoing MUS procedures from 1 January 2017 to 31 December 2021 (*n* = 329).

**Results:**

A total of 279 women (85%) had no complications. Fifty women had one or more complications. Twenty-three women (7%) experienced POUR. Final treatment in 9 women was clean intermittent self-catheterization (CISC). All remained continent. Nine women had the MUS mobilized. This was successful in 8 women who remained continent. Six women had their MUS incised (one after unsuccessful mobilization). Four became incontinent again and 2 remained continent. Eight women had vaginal MUS exposure. Seven attempted recovering of the MUS. This was successful in 3 patients. The remaining had a partial MUS removal. Only 33% remained continent after removal. Ten patients developed de novo urge, but only 2 needed medication.

**Conclusions:**

Mobilization of the MUS must be considered the optimal treatment for POUR when CISC fails. It is the most effective intervention with the best effect on POUR and the lowest risk of incontinence. Concerning vaginal exposure, a trial of recovering should be attempted as the risk of incontinence when undergoing a partial removal of the MUS is considerable.

## Introduction

Stress urinary incontinence (SUI), defined as involuntary leakage of urine caused by physical exertion, sneezing, or coughing [1], is the predominant cause in 30–80% of women with urinary incontinence (UI) [2]. Parity, obesity, and age are risk factors that contribute to development of SUI [3–5]. First-line treatment for SUI is conservative, but in the case of an unsuccessful result, many women opt for surgical procedures.

The midurethral sling (MUS), is in most parts of the world the gold standard for the surgical treatment of SUI [6]. There are different types of MUS, one of them being the tension-free vaginal tape placed via the retropubic route (TVT). The TVT has a high subjective and objective cure rate, ranging from 71 of 97% [2].

The MUS is a safe procedure, but does have some potential complications, both intra- and postoperatively [2, 7]. The most common complications are urgency, hematomas, bleeding, postoperative urinary retention (POUR), infections, and vaginal MUS exposure [8]. The complications are well described, and with a low frequency, but when occurring, international consensus on treatment is lacking [9]. Furthermore, literature on the impact on SUI after the treatment of complications is limited.

With the rise in life expectancy and increased tendency toward obesity combined with the expanded awareness of UI as a disease that might be cured, it can be expected that more women will be needing, and wanting, treatment for SUI. Consequently, it is important to continue monitoring the efficacy of the MUS procedure but also the complications and treatment of these and the impact of the treatment of complications on urinary incontinence.

The main aim of this study was to evaluate the frequency of complications with regard to the MUS procedure with a specific focus on POUR and vaginal exposure, the treatment of these complications, and the potential impact on the efficacy of the MUS procedure.

## Materials and methods

Included in this study are all women undergoing the MUS procedure for SUI at the Department of Obstetrics and Gynecology, Aalborg University hospital during a 5-year period from January 2017 to December 2021. The only MUS system used at the department is the TVT Exact.

Follow-up time varied from 5 years to 6 months. All medical records were followed up to December 2022.

All people living in Denmark are identified by a unique personnel number (CPR). This enables data from different registers to be crosslinked. The women in our study were identified using the national register where all surgical procedures carried out in Denmark are recorded. From this register, all women who had undergone a MUS procedure in our department were extracted. The medical record on each woman was then identified by CPR. The standard medical record, which included the International Consultation on Incontinence Questionnaire Urinary Incontinence Short Form (ICIQ-UI-SF) and the Patient Global Impression of Improvement (PGI-I) completed by the women before and after the TVT procedure were recorded together with baseline health and demographics.

Extracted preoperative information contained demographic data and medical history (Table 1), a description of the physical examination of the pelvic floor, a transvaginal ultrasound, urinary flow examination, examination of residual urine, a cough test, and a 3-day voiding diary. Women with an anamnesis of pure SUI and normal urinary flow with no residual urine, were not investigated with invasive urodynamics.

**Table 1:** Baseline demographics, *N* = 329

Demographics	Data	
Age (years), mean (SD)	52.4	(9.95)
BMI (kg/m^2^), mean (SD)	26.76	(4.89)
< 18.5	2	(0.6)
18.5–24.9	133	(40.4)
25–29.9	117	(35.6)
>30	77	(23.4)
Parity, *n* (%)		
Nulliparous	8	(2.4)
1 vaginal birth	52	(15.8)
2 vaginal births	165	(50.2)
3 vaginal births	77	(23.4)
4 vaginal births	17	(5.2)
>5 vaginal births	6	(1.8)
Surgical delivery, *n* (%)	55^a^	(16.7)
Previous surgery, *n* (%)		
Prolapse	40	(12.2)
Urinary incontinence	12	(3.7)
Hysterectomy (nonprolapse)	29	(8.8)
Other pelvic surgeries	30	(9.1)
Current smoker, *n* (%)	60	(18.2)
Use of alcohol > 7/week, *n* (%)	21	(6.4)
Urinary flow		
Volume (ml) mean (range)	406.45	(40–1,266)
Residual urine, *n* (%)		
<100 ml	325	(99)
Between 100–150	4	(1)
Max flow rate (ml/s), mean (range)	31.8	(10.2–73)

*BMI* body mass index

^a^4 had surgical delivery only

Peri— and postoperative procedures, and any complications, were collected from the medical record. Complications were defined as unintended events occurring during the operation (intraoperative) and postoperative complications as those occurring after the surgical procedure was finished. Re-operation was any surgery taking place after the main MUS procedure but related to the main procedure. If a women developed urge after the MUS procedure, it was categorized as a complication only if it was de novo urgency. Pain was included as a complication, if the woman was seen in the outpatient clinic because of pain, and/or if treatments other than over-the-counter painkillers were needed.

Postoperative urinary retention was diagnosed when the trial of voiding without a catheter (TVOC) was not passed, and was defined as postoperative urinary retention with more than 150 ml in the bladder after a voided volume of at least 250 ml.

All data extracted were entered into the Research Electronic Data Capture (RedCap), which is browser-based, metadata-driven electronic data capture software and workflow methodology for designing clinical and translational research databases.

The study was set up as a retrospective register study carried out for quality assessment. Due to Danish health legislation an approval from the local region (the region of northern Denmark) was needed to access the medical files, ID number F2022-052. No approval was needed from the Danish Ethics Committee or the Institutional Review Board (IRB).

At our department the MUS procedure is performed under local anesthesia amplified with sedation. The procedure is performed as described by Ulmsten [8] via the retropubic route. To guide the tightening of the sling, a cough test is carried out with 300 ml in the bladder. At the end of the procedure the bladder is emptied, and the catheter removed unless there has been a bladder perforation, in which case the catheter is left in for 24 h.

The women are discharged from hospital the same day if they pass their TVOC. If the TVOC is not passed, an indwelling catheter is placed, home nursing arranged, or the women are taught to perform clean intermittent self-catheterization (CISC). The use of catheters ends when the residual urine is less than 100–150 ml.

All women are instructed to contact the personnel at the department if they are insecure regarding bladder emptying after the MUS procedure. The significance of doing so within the first 2 weeks after surgery is expressed very clearly. For women with emptying problems a phone follow-up is planned on the first postoperative day, and a control follow-up is planned. For women with persistent POUR, the MUS is mobilized within the first 2 weeks postoperatively.

Mobilization of the MUS is done under general anesthesia. The vaginal incision from the MUS procedure is reopened. The sling is loosened from the vaginal wall with a small péan. Two small péans are placed as lateral as possible (avoiding pulling in the midline) on the MUS. A gentle traction on the MUS is then performed by pulling the Spencers, and the tape is mobilized about 2–5 mm.

If mobilization of the MUS is insufficient, an incision of the sling can be performed. This is also done if a woman is seen too late for sling mobilization. Incision of the tape is done under general anesthesia. The vaginal incision is reopened, the sling is loosened from the vaginal wall, and the MUS incision made.

All women can contact the nurses specialized in urogynecology during office hours with questions regarding the MUS procedure and the postoperative period. All women have a routine telephone follow-up 3 months after the MUS procedure. The PGI-I and ICIQ-UI-SF are reported at this interview. If all is well, the women are discharged from the department. If there are still issues regarding POUR, incontinence, or newly occurring symptoms after surgery, the women are seen in the clinic.

## Results

In total, 329 women were identified and included. Out of these, 85% (279 out of 329) had no complications at all. Fifty women had one or more complications. A total of 90 complications were noted among the 50 women (Table 2).

**Table 2:** Peri- and postoperative complications

Type of complication	Number (%) of women with complications	Number (%) of women needing an operation for the complication
Intraoperative complications		
Bladder perforations	5 (1.5)	–
Postoperative complications		
Infections	16 (4.9)	
- rUTI	12 (3.6)	5^a^
- Tissue	4 (1.2)	–
Hematoma	3 (1)	–
Bleeding	–	–
Pain	6 (1.8)	4^b^
Exposure to vaginal mucosa	8 (2.4)	8
Persistent SUI	3 (0.9)	3^c^
POUR >24 h	23 (7.0)	14
De novo urgency	10 (3.0)	–

*rUTI* recurrent urinary tract infection, *SUI* stress urinary incontinence, *POUR* postoperative urinary retention

^a^1 also had sling erosion and sling removal. 4 also had POUR and sling incision

^b^2 also had sling erosion

^c^1 also had sling erosion, and 1 had POUR. Both underwent sling removal. 2 of the 3 underwent bulking

**Fig. 1:**
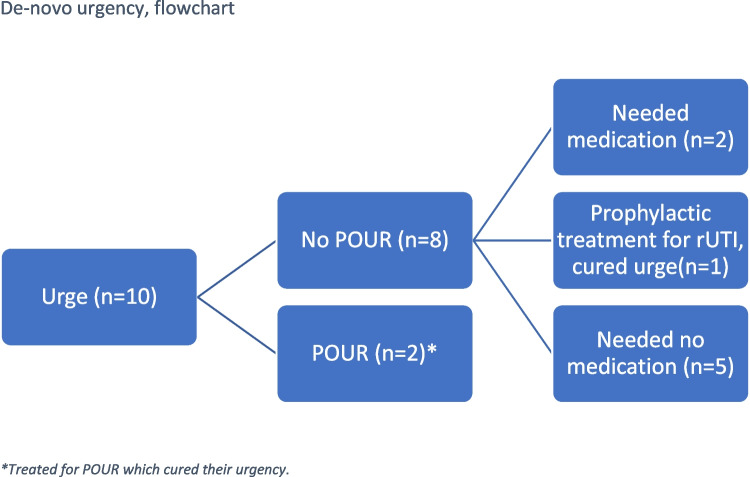
De novo urgency, flowchart. *Asterisk* = treated for postoperative urinary retention (*POUR*), which cured their urgency. *rUTI* recurrent urinary tract infection

Ten of the women developed de novo urgency (3.0%). Five of them did not feel the urge so disturbing that they needed medical treatment. Two were treated medically. One also had rUTI and after initiation of prophylactic treatment for this, her urgency ceased. The last two women, in addition to de novo urgency, also developed POUR, for which they needed further treatment (Fig. 1).

**Fig. 2:**
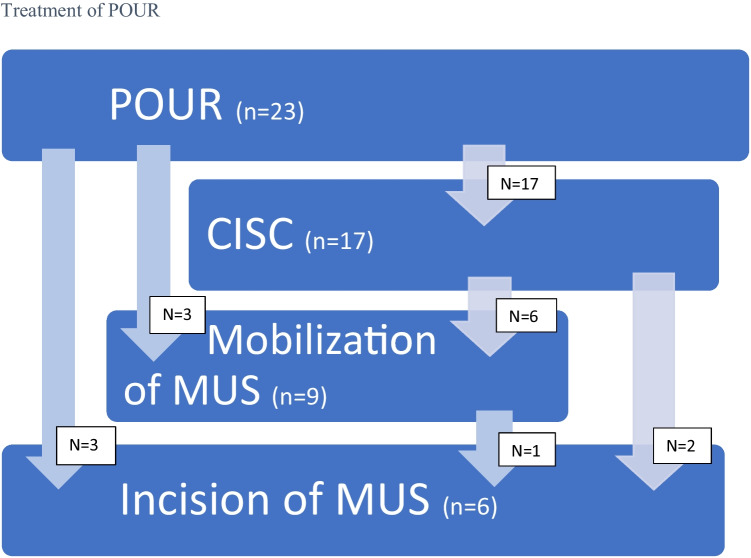
Treatment of postoperative urinary retention (*POUR*)

Postoperative urinary retention was the most common complication (*n* = 23; Fig. 2). Nine of these women were treated only with the use of a catheter (either CISC, catheterization performed by home nurses or an indwelling catheter) and could after a few days terminate the use of catheters, experiencing trouble-free voiding. They were all continent (Table 3).

**Table 3:** Treatment of complications and impact on flow and incontinence

Complication	Final treatment	Number of women	Urinary flow after treatment	Continent yes/no/partly
POUR	CISC	9	Normal	9 Yes
	Mobilizing of MUS	8	Normal	8 Yes
	MUS incision	6	3 Normal	2 Yes
			3 Not normal	3 Partly
				1 No
Tape erosion	Recovering of MUS	2	1 Normal	2 Yes
			1 Not normal	
	Removal (partly) of MUS^a^	6	6 Normal	2 Yes
				1 Partly
				3 No

*POUR* postoperative urinary retention, *CISC* clean intermittent self-catheterization, *MUS* midurethral sling,

^a^One was due to pain after successful recovering

Nine women were treated with mobilization of the MUS, on average 9.9 (6–14) days postoperatively. For 8 of them mobilization was sufficient treatment. POUR was cured, and the women were still continent. For 1 woman mobilization was insufficient, and her MUS was subsequently incised. The average follow-up time for the 9 women is 63.3 months. During follow-up time 2 of the women have had their sling incised, but none of the other 7 have had renewed contact with the department owing to SUI relapse or complications with regard to the MUS (Table 4).

**Table 4:** Data on events in the follow-up period and satisfaction among the women based on International Consultation on Incontinence Questionnaire Urinary Incontinence Short Form and Patient Global Impression of Improvement for the group of women undergoing midurethral sling (*MUS*) mobilization

Woman	Incontinence scores before surgery	Incontinence scores 3 months after surgery	Comparison of outcome 3 months after MUS operation	Follow-up time from main MUS operation (months)	Events during the follow-up period
1	16	0	Much better	61	After a car accident MUS exposure and incontinence. Successful covering and bulking. Developed pain and sling partly removed
2	18	0	Much better	54	Experienced troublesome voiding after labor complicated by a large amount of residual urine. Still continent
3	–	0	Much better	57	No problems during follow-up period
4	16	–	–	69	No problems during follow-up period
5	17	14	A little better	72	Worsening in urgency, insufficient treatment for SUI. MUS incised
6	16	0	Much better	39	No problems during follow-up period
7	16	–	–	72	No problems during follow-up period
8	16	0	Much better	68	No problems during follow-up period
9	15	0	Much better	78	No problems during follow-up period

*SUI* stress urinary incontinence

In total, 6 women needed incision of the MUS. One had had an unsuccessful mobilization. Two of the women had performed CISC initially after the MUS procedure, but passed their TVOC within the first 14 days, and were discharged. They were re-referred months later. Both had developed de novo urge, in addition to POUR. For one of the women, the urge was described as nondisabling, but she needed to perform CISC when she had a very full bladder. She could not manage CISC on her own and wanted an MUS incision. The other woman had her urgency unsuccessfully treated with medicine and an invasive urodynamic examination was performed. It showed residual urine and obstructive flow and the decision to make the MUS incision was taken. Both women normalized their urinary flow after the incision. One was still continent, the other only partly (Table 3).

Three of the women went directly to incision of the tape, as they were seen too late for mobilization. One never contacted the department and her problem was relieved at the routine follow-up, 3 months postoperatively. For 1 woman a language barrier led to a misunderstanding of her voiding dysfunction. The last woman contacted the department after 14 days, but for reasons unknown, no action was taken until the 3-month follow-up. The result of the incision for 2 of these women was a better but not normal urinary flow. One was continent and the other only partly continent. The last woman regained a normal urinary flow but was incontinent afterwards. She was treated successfully with an incontinence pessary (Table 3).

Eight women (2.4%) had vaginal MUS exposure. One of the women was diagnosed 28 months after her TVT procedure. She was re-referred to the department because of urgency and rUTI. During the examinations, the MUS exposure was found. A urodynamic examination showed obstructed flow, so no attempt was made at sling covering. Instead, a partial sling removal was carried out. She had a relapse of her SUI but regained a normal urinary flow. She later received bulking as a treatment for her SUI.

The other 7 women all underwent an attempt to cover the MUS. One woman had the procedure done twice. For 3 of the women the procedure was successful, and the sling did not re-extrude. However, 1 did later develop pain, and the sling was partially removed 22 months after the MUS procedure (7 months after treatment of the sling exposure). This woman had originally undergone a successful mobilization of the sling 14 days after the main MUS procedure. She had been discharged but was re-admitted after a car accident as her UI returned. Here, the vaginal MUS exposure was diagnosed. The pain disappeared with partial removal of the MUS.

For the woman who had the covering procedure done twice, and for 3 other women, the covering was unsuccessful. The MUS re-extruded, and they all underwent partial removal of the sling, on average 12.5 months after the main MUS procedure. In total, only 33% (2 out of 6) stayed continent after partial removal of the sling compared with 100% with sling covering (Table 3). The ICIQ incontinence scores were obtained 3 months postoperatively. The ICIQ data are therefore not applicable for a satisfactory result. The incontinence status was obtained at a final control where the ICIQ was not used.

Two women had their MUS partially removed owing to pain. One is described above. The other woman also had pain before her MUS procedure but felt that it had worsened. She also developed de novo urgency and had no relief on urgency medication. Her sling was partially removed, and her SUI returned. The pain close to her urethra disappeared, but she continued to have lower abdominal pain. Six women complained of pain but was this was only the reason for partial tape removal twice. Among the 4 others, pain disappeared spontaneously in 2, after physiotherapy in 1, and the last 1 was lost to follow-up.

Other complications were perioperative bladder perforations, infections, and development of hematomas, but none of these led to further surgery (Table 2).

In total, 7% of all women (*n* = 23) had further surgery after the MUS procedure. Six women had surgery twice after the main surgery, 1 had three and 1 had four additional operations. This was a total of 34 extra operations among all 329 women.

## Discussion

Postoperative urinary retention, de novo urgency, and vaginal sling exposure were the most common complications with regard to the MUS operation. Urgency alone did not give rise to any further operations. Exposure and POUR were the two complications that most often led to further operations.

The treatment for persistent POUR with mobilization of the MUS was found to be uncomplicated. It had a good outcome with a normalizing of the urinary flow and unaltered continence (Table 3). When incising the sling, data were not as promising. Only 33% (2 out of 6) remained continent, and only 50% normalized their urinary flow. This is in line with a Canadian study showing that 78% of women with POUR undergoing incision of their sling became incontinent again, whereas none of those who underwent mobilization became incontinent [10] and a French study concluding that early mobilization of the MUS in the case of POUR offers better efficacy than delayed mobilization, with a lower risk of recurrent/persistent SUI [11]. A Norwegian study comparing CISC, sling mobilization, and incision also found that women who had their sling mobilized or who had intermittent catheterization, scored better on all postoperative outcomes than those who had a sling incision (*p* < 0.001). Sling incision was needed more often after intermittent catheterization than after mobilization (*p* = 0.023) and those undergoing sling mobilization had a negative stress test significantly more often (*p* = 0.033) and were more often "very satisfied" with the treatment (*p* = 0.006) than those who were primarily catheterized [12].

Vaginal MUS exposure is a less common complication, but also more complicated to treat. The complication rate in this study is comparable with that of other studies [13–15]. It can occur shortly after the operation but can also be seen much later. For just over 40% of the women, re-suturing the vaginal wall over the sling was successful and the women did not develop incontinence. The results regarding incontinence after partial removal are worse and comparable with when the sling is incised, with about one third remaining continent. In this study no relation concerning time between the main MUS procedure, the partial removal of the MUS, and the recurrence of urinary incontinence was observed.

The collected data were compared with earlier data on MUS complications in our department. The number of complications has remained stable over the last 15 years [16, 17]. Only three surgeons at a time have been performing the MUS procedure in our department. In this way, the volume has been kept high for the surgeons, which has previously been shown to reduce complications [18, 19]. Over time, we have employed the same procedure to treat POUR and vaginal sling exposures [16, 17].

One limitation of the study is the retrospective design. Because of this design, some data may be missing, incomplete, or incorrect. However, the culture of filling in medical records is very strong, and data reported in the medical records are considered true. As an insurance of the data entered into the medical records, a large amount of the data also comes from archives where documents with data on the ICIQ-UI-SF and PGI-I are scanned in directly, and hence not interpretated by health personal, but they do, in schematic form, consist of the woman’s own description. These questionaries are validated. The only women with MUS complications not enrolled in the study are those who have moved to a different region in the country after the 3 months’ follow-up on the MUS procedure. This is expected to be a very small number.

The number of women in the study is also a limitation. The challenge is that the number of complications are low, and hence a very large number of women would be needed to accumulate a large amount of complications and their treatment. In our department we previously analyzed data on the MUS procedure and its complications. For this reason, we can see that the rate of complications is stable, and the treatment sufficient.

The variation in follow-up and the short follow-up time for some of the women are also limitations. In general, POUR is diagnosed within the first 3 months, but MUS exposure may occur later. With POUR being the most common complication, a treatment of POUR is what is most often needed. Despite the variable follow-up period, this study does provide evidence-based data describing the treatment of POUR after MUS.

To reduce the risk of variation in interpretating data collected in the records and archive, all data have been collected by only two people. If in doubt with regard to any data, the records were looked through by both data collectors, and hence data were as aligned as possible.

Regarding complications on the MUS procedure, our data are comparable with previously reported data [12, 15, 19]. The chosen treatment for the complications is also in line with these data. We did not look at the cost–benefit regarding the treatment of complications, but Vargas Maldonado et al. found a better cost–benefit when doing an early loosening of the sling than when performing a late incision [20].

With only 23 of the women (7%) having repeated surgery because of complications, the MUS procedure can still be considered a safe procedure with few complications.

We find that for vaginal MUS exposure, recovering should be performed despite a success rate under 50%, as partial removal of the tape causes relapse of incontinence for about 70% of the women.

Due to our data, we find that if POUR is diagnosed early, it can almost always be sufficiently treated by mobilizing the sling, and the efficacy of the MUS procedure can be retained. It can therefore be reasoned that information to all women on POUR and the importance of returning within the first 14 days after the MUS procedure, if in any doubt, is of the greatest significance.
